# Neoadjuvant chemotherapy, DEB-TACE, and 3D-printed prosthesis for primary pelvic pleomorphic undifferentiated sarcoma: a case report

**DOI:** 10.3389/fonc.2025.1641058

**Published:** 2025-12-09

**Authors:** Jiahui Liu, Jiawen Wang, Hui Dai, Qi Feng

**Affiliations:** Department of Orthopedics, The Fourth Hospital of Hebei Medical University, Shijiazhuang, Hebei, China

**Keywords:** primary pelvic undifferentiated sarcoma, drug-eluting bead transarterial chemoembolization, 3D-printed prosthesis, neoadjuvant chemotherapy, case report

## Abstract

Pleomorphic undifferentiated sarcoma (PUS) is a highly malignant soft tissue sarcoma typically found in the extremities or visceral organs, with primary pelvic cases being exceedingly rare and lacking standardized management strategies. We present the case of an elderly female with primary pelvic PUS who presented with right hip pain and progressive immobility. The tumor was large and involved surrounding tissues, making single-stage en bloc resection unfeasible. A multimodal strategy was implemented, including neoadjuvant chemotherapy combined with anlotinib, followed by drug-eluting bead transarterial chemoembolization (DEB-TACE), and subsequent resection and reconstruction with a 3D-printed pelvic prosthesis were performed. The patient achieved satisfactory functional recovery, and at the 12-month follow-up, there was no evidence of recurrence or metastasis and tumor was judged to have achieved pathological complete remission (pCR).This case demonstrates the feasibility of individualized multimodal therapy for complex pelvic PUS and provides valuable insights for managing this challenging entity.

## 1 Introduction

Pleomorphic undifferentiated sarcoma (PUS) is a rare and highly aggressive soft tissue malignancy characterized by a poor prognosis, manifesting as a low five-year survival rate and a high rate of distant metastases that occur in approximately 40% of patients (1, 2). Primary PUS in the pelvis presents significant surgical challenges due to its proximity to vital structures, often resulting in high rates of postoperative complications and recurrence. The lack of standardized treatment guidelines necessitates innovative, multimodal approaches to improve outcomes (3).

Conventional treatment strategies, including surgery, radiotherapy, and chemotherapy, have limited effectiveness for PUS and often fail to achieve satisfactory outcomes. Complete tumor resection is frequently unachievable. In recent years, advancements in neoadjuvant chemotherapy, drug-eluting bead transarterial chemoembolization (DEB-TACE), and 3D-printing technology have provided new therapeutic opportunities for pelvic PUS. Neoadjuvant chemotherapy can reduce tumor size and surgical difficulty; DEB-TACE enhances intratumoral drug delivery while minimizing intraoperative blood loss; and personalized 3D-printing technology enables surgeons to better visualize tumor morphology and location, facilitating precise tumor resection (4).

Here, we present a rare case of primary pelvic PUS that was successfully managed using a novel multimodal strategy combining neoadjuvant chemotherapy, drug-eluting bead transarterial chemoembolization (DEB-TACE), and reconstruction with a 3D-printed prosthesis. This specific combination of therapeutic modalities is rare in clinical practice and has not been systematically reported in China. By detailing this comprehensive treatment protocol and its successful outcome, we aim to highlight the potential of this integrated approach to achieve local disease control and functional reconstruction in complex sarcoma cases, and to provide a valuable reference for the management of this challenging condition.

## 2 Case presentation

**Figure 1:**
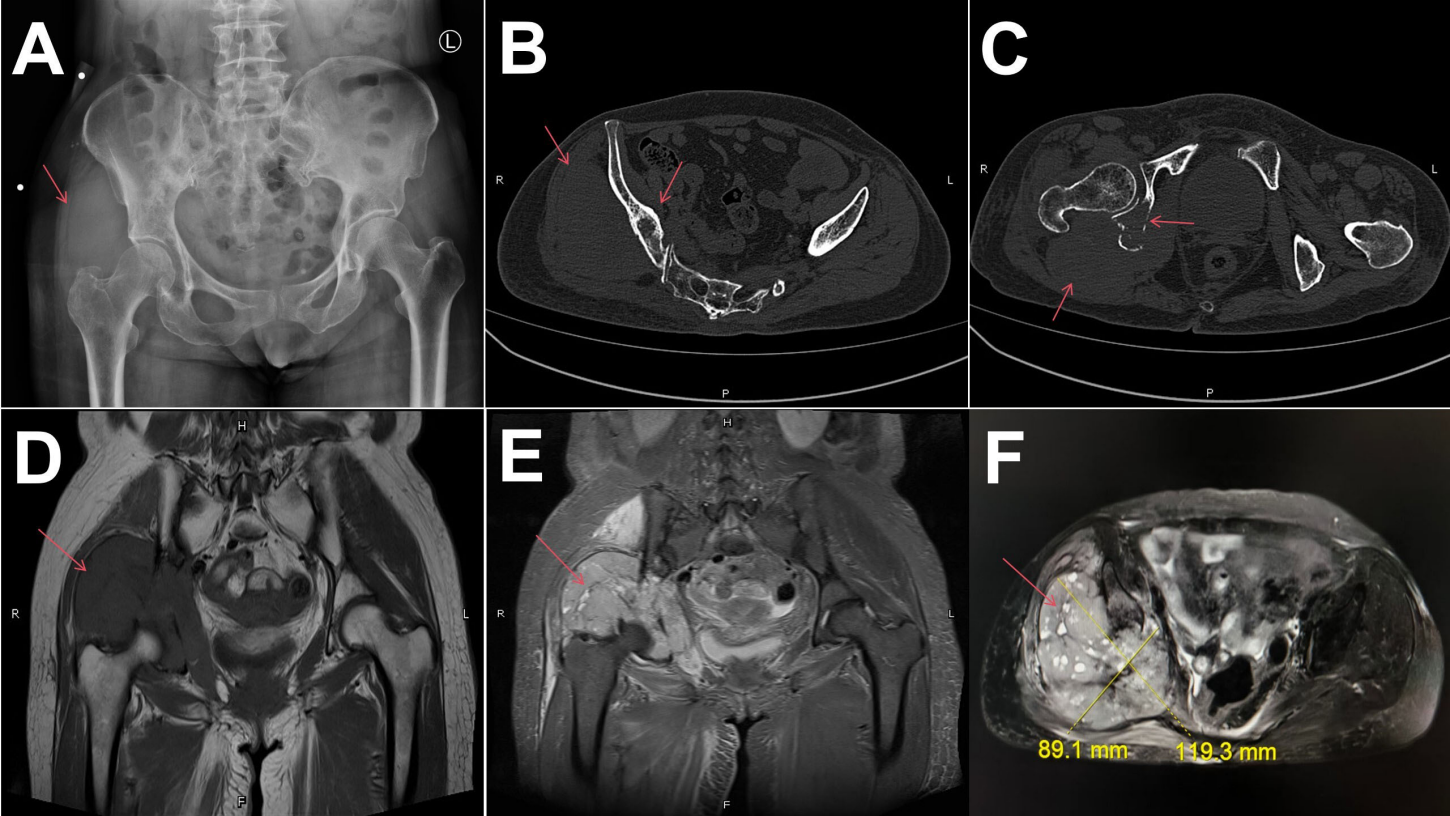
Preoperative radiological findings. **(A–C)** X-ray and CT before operation:showed a lytic lesion in the right iliac bone and hip joint bone with cortical destruction, surrounding soft tissue mass. **(D–F)** MRI before operation: compared with muscle tissue, showed isointense on T1WI and slightly hyperintense on T2WI, with scattered nodular hyperintensity. The adjacent gluteus and iliopsoas muscle were affected.

A 63-year-old female patient was admitted to our hospital with a four-month history of right hip pain, which had progressively worsened over the past month, rendering her unable to walk. Physical examination revealed a hard, poorly mobile, and large soft tissue mass measuring about 30cm × 20cm × 10 cm in the right hip region. The overlying skin was tense without ulceration or superficial varicosities. Joint mobility was restricted, and the Patrick’s sign was positive. X-ray and computed tomography (CT) of the hip showed bone destruction in the right ilium and hip joint, along with a surrounding soft tissue mass suggestive of malignancy (**Figures 1A–C**). Magnetic resonance imaging (MRI) of the pelvis revealed an abnormal signal in the right ilium, ischium, and acetabulum, with extension into the surrounding gluteal and iliopsoas muscles (**Figures 1D–F**).

**Figure 2:**
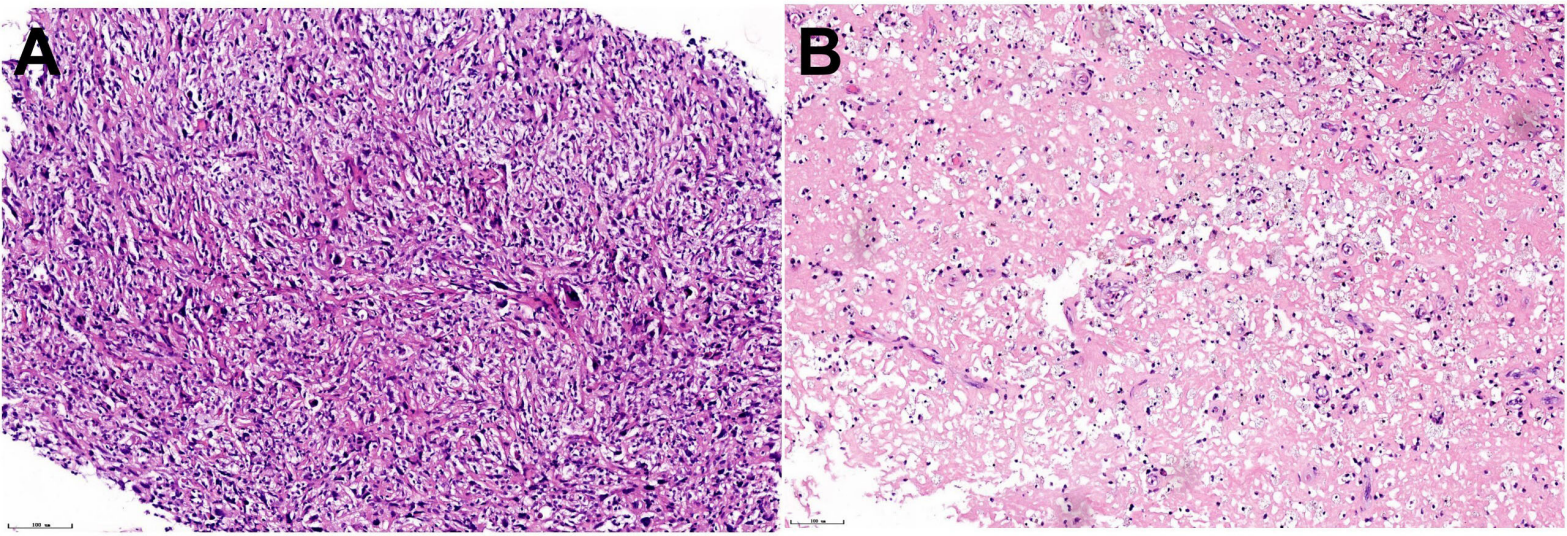
Histopathological examination of the tumor. **(A)** Under the microscope, spindle-shaped tumor cells are densely arranged, varying in size, lacking differentiation, and exhibiting significant atypia. The nuclei are large and deeply stained, with prominent nucleoli. Numerous pathological mitotic figures were observed, and the chromatin showed uneven thickness. Additionally, fibroblast cells, histiocyte-like cells, varying numbers of multinucleated giant cells, chronic inflammatory cells, and foam cells were present, consistent with undifferentiated sarcoma. (HE ×200) **(B)** The nuclei of tumor cells vary in size and exhibit irregular shapes. Some of the nuclei display signs of apoptosis, such as chromatin condensation or nuclear fragmentation. The cytoplasm shows vacuolization or eosinophilic change, conform to changes after chemotherapy. (HE×200).

Ultrasound-guided biopsy confirmed a diagnosis of PUS, characterized by spindle-shaped undifferentiated tumor cells with significant atypia, hyperchromatic nuclei, prominent nucleoli, and active mitosis under the optical microscope (**Figure 2A**). Immunohistochemistry results were as follows: AE1/AE3 (-), CD68 (+), Ki67 (30%), Des (-), Calponin (+), CD163 (+), S100 (-), CD34 (-), SOX-10 (-), and P40 (-). Chest and abdominal CT and whole-body bone scans showed no evidence of distant metastases. According to the Enneking staging system, the tumor was classified as stage IIB. Due to the extensive involvement of the surrounding tissues, achieving wide-margin resection in a single surgery was deemed impractical.

**Figure 3:**
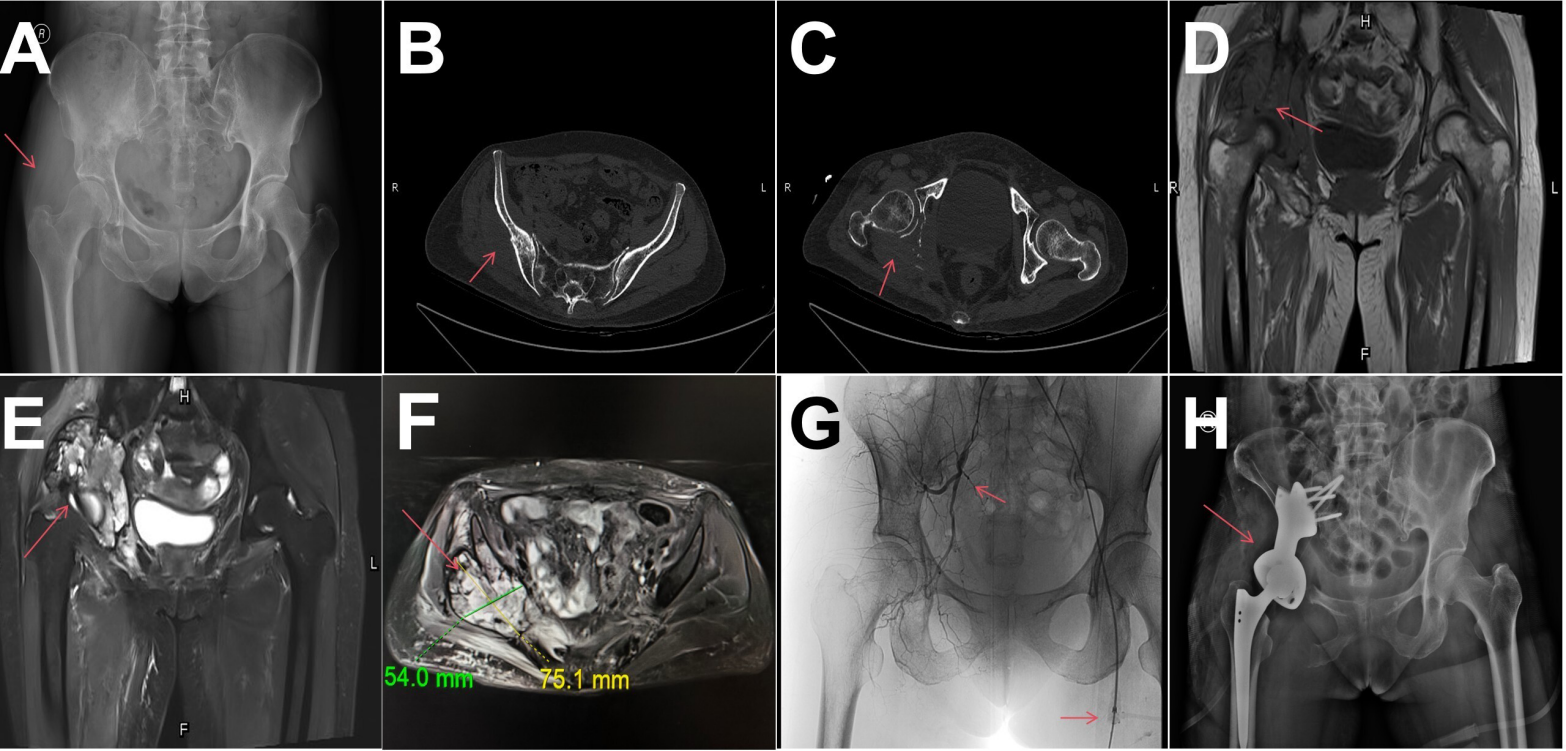
Post-chemotherapy imaging and surgical intervention. **(A-F)** X-ray, CT and MRI after chemotherapy: showed a significant reduction in tumor volume compared to preoperative images. **(G)** The vascular supply to the tumor was revealed by DEB-TACE. **(H)** X-ray after operation: the fixation of the 3D-printed prosthesis is satisfactory, showing no signs of loosening.

The patient underwent three cycles of neoadjuvant AI chemotherapy (liposomal doxorubicin 20 mg on days 1–3 and ifosfamide 2 g on days 1–5, administered intravenously every three weeks). During the chemotherapy intervals, she received two cycles of oral anlotinib (10 mg daily on days 1–14 of a three-week cycle). After treatment, her pain improved, and the tumor size was significantly reduced, with a softer texture. Imaging studies, including X-ray, CT, and MRI, indicated significant tumor shrinkage. (**Figures 3A–F**) During this combination therapy, the patient developed adverse events including neutropenia, thrombocytopenia, and intractable vomiting. These symptoms resolved with appropriate supportive management, and the patient’s condition remained stable thereafter.

To reduce the risk of tumor recurrence and minimize intraoperative blood loss, DEB-TACE was performed (**Figure 3G**). Under local anesthesia, the Seldinger technique was used to insert an arterial sheath via the left femoral artery. Angiography confirmed that the tumor was supplied by the right inferior gluteal artery. Drug-eluting beads (100–300 μm) loaded with 40 mg pirarubicin were used for transarterial chemoembolization of the feeding artery. Post-embolization angiography showed complete disappearance of tumor staining. The total radiation dose during the procedure was 159 mGy.

Preoperative planning involved creating a 3D-printed model of the pelvis to design an osteotomy guide and pelvic prosthesis. A modified ilioinguinal incision extending posteriorly along the iliac crest was utilized for adequate exposure. During surgery, the tumor was completely resected, and a 3D-printed pelvic prosthesis was implanted under general anesthesia (**Figure 3H**). The postoperative course was generally uneventful with no wound complications, infections, or need for reoperation, except for gross hematuria one week after surgery, cystoscopy confirmed hemorrhagic cystitis, likely caused by ifosfamide. Symptomatic treatment resolved the hematuria within five days. The patient was able to stand and ambulate with walker assistance under weight-bearing restrictions 12 days postoperatively. Postoperative pathological examination under the optical microscope revealed extensive hemorrhagic necrosis with associated foam cell and histiocytic reactions. In the surrounding stroma, scattered apoptotic figures—exhibiting chromatin condensation and nuclear fragmentation—as well as cytoplasmic vacuolization and eosinophilic changes were observed, consistent with a profound treatment effect. Based on RECIST 1.1 criteria, the therapeutic response was evaluated as Pathological Complete Response (PCR) (**Figure 2B**).

**Figure 4:**
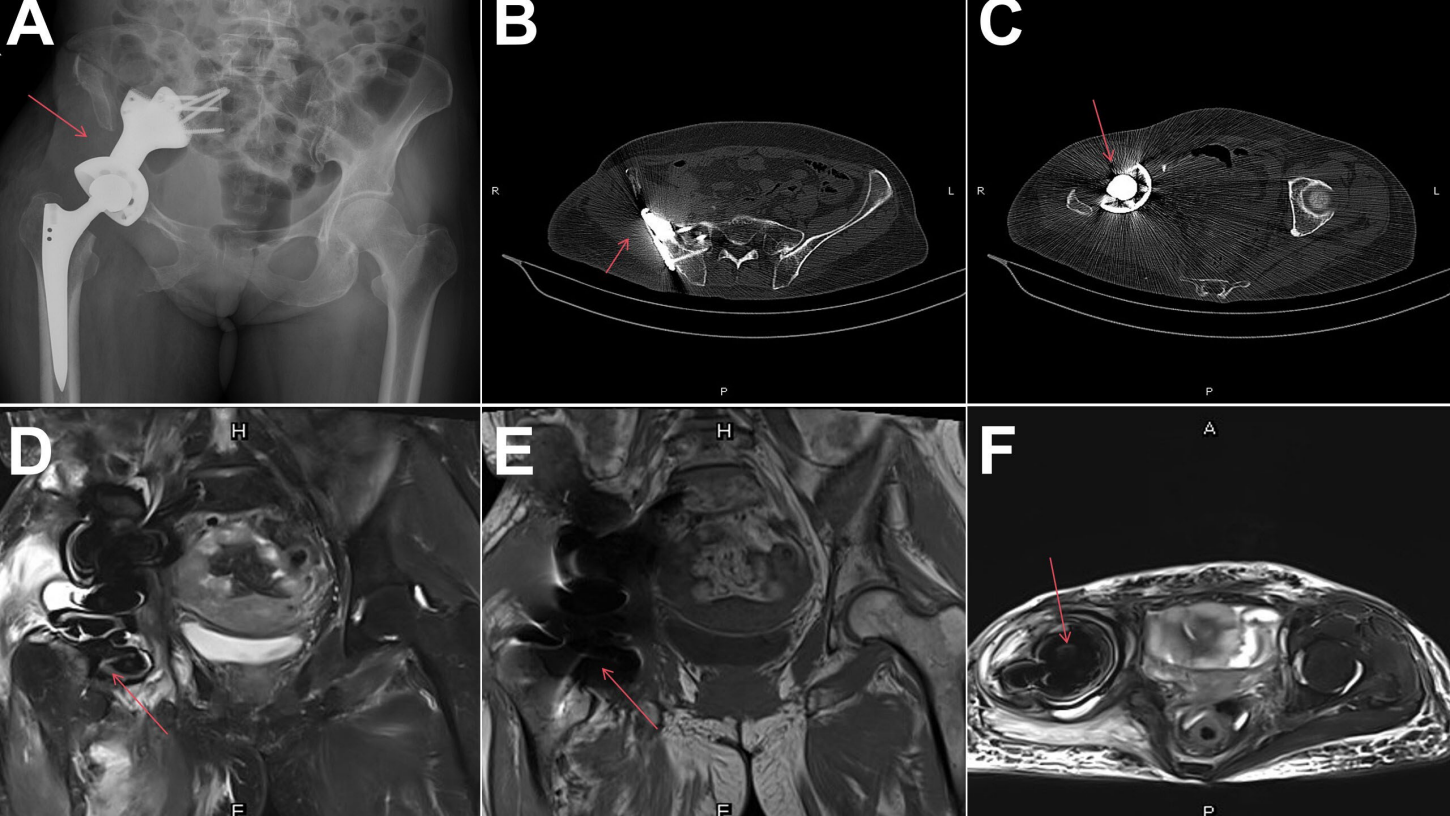
Twelve-month follow-up imaging. **(A–F)** Follow-up X-ray, CT, and MRI scans after 12 months: no signs of recurrence or metastasis were observed.

One cycle of postoperative chemotherapy with liposomal doxorubicin was administered, but severe adverse events, including grade IV neutropenia, grade III thrombocytopenia, and intractable vomiting, prompted the patient to discontinue further chemotherapy. At the 12-month follow-up, the patient had good limb function, with a Musculoskeletal Tumor Society (MSTS) score of 20. Imaging studies showed that the prosthesis was well-positioned, with no signs of tumor recurrence or distant metastasis (**Figures 4A–F**).

**Figure 5:**
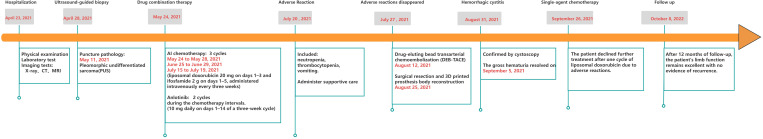
Flowchart. This figure depicts the relevant workflow and critical timeline of the case.

The relevant workflow for the aforementioned case is illustrated in **Figure 5**.

## 3 Discussion

The management of primary pelvic pleomorphic undifferentiated sarcoma (PUS) necessitates innovative, multimodal strategies due to its rarity, large size at presentation, and complex anatomical location. This highly malignant soft tissue sarcoma was first described in 1964 as malignant fibrous histiocytoma (MFH) (5).It predominantly affects middle-aged and elderly adults, with a distant metastasis rate of approximately 32.5% and a local recurrence rate of 22.9%. The 5-year overall survival rate is around 76.4%. Surgical margin status (R0 vs. R1/R2) represents the most significant independent prognostic factor (6). Due to its uncertain histological origin and lack of specific immunohistochemical markers, it is now uniformly classified as PUS (7). Clinically, PUS can arise in any part of the body, with most cases presenting as deep-seated, rapidly growing, painless masses. The most common sites involved are the extremities, followed by the trunk (8). Primary PUS in the pelvis, however, has only been documented in case reports (9, 10).

NAC is typically recommended for STS with large tumors, those invading critical organs, and tumors closely associated with vital nerves or vessels. It is particularly valuable when achieving negative surgical margins or preserving limb function is challenging (11, 12). This approach is supported by evidence, such as a retrospective study of 356 patients with high-grade limb STS—over half of whom had PUS—which demonstrated that neoadjuvant chemotherapy with the AIM (adriamycin, ifosfamide, and mesna) regimen significantly improved survival, especially in patients with tumors larger than 10 cm (13). A key development in optimizing neoadjuvant regimens has been the introduction of liposomal formulations. Compared to conventional doxorubicin, pegylated liposomal doxorubicin (PLD), a polyethylene glycol-encapsulated formulation of doxorubicin, offers several advantages, such as prolonged half-life, higher drug concentration in tumors, and slower drug release (14).These properties translate into an improved safety profile, with studies confirming reduced rates of bone marrow suppression, vomiting (15), and notably, cardiotoxicity (16, 17). Importantly, the efficacy of PLD in STS is well-established (18–20), with potential for long-term remission even in patients who failed prior doxorubicin-based therapies (21). This evidence makes PLD a viable component in modern neoadjuvant strategies. Beyond chemotherapy optimization, the combination of targeted agents like anlotinib represents another promising avenue. Anlotinib, a small-molecule anti-angiogenic tyrosine kinase inhibitor, has shown promising results in advanced, refractory STS, including PUS. Among 166 patients with advanced STS, including 19 with PUS, anlotinib yielded progression-free rates of 58%, 36%, and 14% at 12, 24, and 36 weeks, respectively, with a median progression-free survival (PFS) of 4.1 months (22). The rationale for combining it with chemotherapy is strengthened by preclinical evidence; in a patient-derived xenograft (PDX) mouse model of PUS, anlotinib alone or epirubicin alone significantly inhibited tumor growth, while the combination of high-dose anlotinib and epirubicin demonstrated the most pronounced tumor suppression (23). Clinically, a retrospective study further revealed the efficacy of anlotinib combined with PLD in treating advanced STS. Most adverse events (AEs) were mild and tolerable (24). In this case report, following combination therapy with anlotinib and neoadjuvant chemotherapy, the tumor responded well, This satisfactory therapeutic effect in a rare case of primary pelvic PUS highlights the potential utility of this novel combination for achieving tumor downstaging in complex anatomic sites; Nevertheless, the application of anlotinib specifically combined with chemotherapy as neoadjuvant therapy (NAC) for unresectable soft tissue sarcoma (STS) remains a sparsely documented and fraught with challenges.

The challenges associated with this novel regimen were underscored in our patient by the considerable systemic toxicity that arose despite its therapeutic efficacy. Notably, even with the optimized neoadjuvant backbone incorporating pegylated liposomal doxorubicin (PLD) to mitigate classic anthracycline toxicities, our patient nevertheless experienced significant adverse events, such as hemorrhagic cystitis, severe neutropenia, thrombocytopenia, and intractable vomiting, requiring careful monitoring and dose management.

Hemorrhagic cystitis (HC), observed postoperatively in our case, is primarily associated with alkylating agents such as ifosfamide. Its active metabolites, particularly acrolein, accumulate in the bladder, inducing direct mucosal irritation and bleeding (25). Although it is not yet clear whether anlotinib monotherapy is independently associated with HC, its anti-angiogenic properties may theoretically exacerbate mucosal ischemia and impair repair, potentially potentiating chemotherapy-induced injury (26). This underscores the necessity of rigorous uroprotective strategies, including continuous hydration and mesna co-administration, complemented by frequent urinalysis throughout treatment (27).

Profound myelosuppression—manifesting as grade IV neutropenia and grade III thrombocytopenia—was another major challenge in our patient. This can be attributed to the synergistic toxicity of the combined chemotherapeutic and targeted agents. Ifosfamide metabolites cause DNA cross-linking, while doxorubicin induces double-strand breaks, collectively triggering p53-mediated apoptosis in highly proliferative bone marrow cells (28, 29). Anlotinib likely directly suppresses bone marrow hematopoiesis by inhibiting key signaling pathways crucial for the proliferation, differentiation, and survival of hematopoietic stem/progenitor cells. It may also disrupt the bone marrow microenvironment by targeting receptors such as VEGFR, thereby inhibiting angiogenesis within the bone marrow. Clinical evidence supports this interaction: in a study of 103 patients receiving doxorubicin, ifosfamide, and cisplatin, grade 3–4 neutropenia and thrombocytopenia occurred in 90% and 47% of participants, respectively (30). In a study of 55 patients with advanced hepatocellular carcinoma treated with anlotinib hydrochloride, neutropenia occurred in 14.55% of patients, while thrombocytopenia was observed in 20% (31). Furthermore, a meta-analysis incorporating 16 studies with 1,083 patients revealed that the addition of anlotinib to chemotherapy significantly increased the risk of myelosuppression (32). Therefore, neutropenia warrants prompt antibiotic prophylaxis or therapy to avert or control infection, with granulocyte colony-stimulating factor added in severe cases, whereas thrombocytopenia demands meticulous bleeding precautions and close platelet monitoring, accompanied by immediate platelet transfusion when indicated.

Gastrointestinal toxicity, including intractable vomiting, likely resulted from both chemotherapy and anlotinib inhibiting the regeneration and repair of the gastrointestinal mucosa and compromising the mucosal barrier, albeit the latter is less emetogenic. Effective control requires combination antiemetic therapy with 5-HT_3_ and NK-1 receptor antagonists (33).

Finally, the potential for impaired wound healing and increased intraoperative bleeding associated with anti-angiogenic agents (34) directly influenced our clinical decision-making. For this reason, we withheld the third cycle of anlotinib. This dilemma—balancing tumor progression risk during drug interruption against surgical safety—highlights an ongoing challenge in sarcoma therapy. Some studies have explored combining anti-angiogenic agents with radiotherapy in the neoadjuvant setting to mitigate this risk (35, 36), suggesting a potential avenue for future protocol optimization.

Despite achieving marked tumor downstaging, the combined regimen was limited by significant treatment-related toxicities, which necessitated proactive supportive management. These challenges, compounded by the inherent complexity of integrating anti-angiogenic therapy with surgical scheduling, underscore the necessity of refined patient selection criteria and optimized treatment protocols. Future efforts must prioritize toxicity mitigation strategies and highly individualized therapeutic scheduling to maximize clinical efficacy while minimizing adverse outcomes.

To mitigate the significant risk of intraoperative hemorrhage associated with this highly vascular pelvic tumor and to enhance local cytoreduction, we employed DEB-TACE as a novel neoadjuvant strategy. This decision deliberately expands upon the conventional role of embolization, which has historically been relegated to a purely preoperative hemostatic adjunct (37, 38). Angiographic evaluation precisely identified the inferior gluteal artery as the dominant feeding vessel, which was then super selectively embolized with pirarubicin-loaded drug-eluting beads. While transarterial chemoembolization (TACE) is a well-established cornerstone for malignancies such as hepatocellular carcinoma (39), the application of DEB-TACE for unresectable soft tissue sarcomas (STS) remains profoundly under investigated, representing a significant literature gap (40). The prevailing literature largely overlooks its potential as a cytoreductive and drug-delivery modality beyond mere hemodynamic modification. Our case directly challenges this narrow paradigm by demonstrating the successful integration of DEB-TACE within a multimodal protocol, achieving synergistic local tumor control and surgical risk mitigation. The favorable treatment response observed provides compelling evidence that DEB-TACE can function as a potent locoregional therapeutic approach even in non-visceral sarcomas.

Having established a surgical window through successful cytoreduction via the multimodal neoadjuvant approach, we confronted the subsequent challenge of reconstructing the resultant massive pelvic defect, particularly given the complex biomechanical demands of the acetabular region. Traditional methods of pelvic reconstruction, such as saddle prostheses or custom screw-rod systems, are often hampered by difficulties in achieving accurate acetabular component alignment and rotation center restoration, frequently leading to suboptimal functional outcomes, gait abnormalities, and compromised long-term implant stability (41–43). The advent of 3D printing technology has revolutionized this field. Preoperative 3D-printed models enable the creation of customized osteotomy guides, facilitating precise resection while preserving as much healthy bone as possible. Using additive manufacturing principles, patient-specific prostheses can be tailored to anatomical structures, achieving precise reconstruction of bone defects (44–46). Preoperative simulation surgery on 3D-printed models can reduce intraoperative reconstruction time (47). Additionally, preplanned screw channels allow precise fixation of screws through the sacroiliac joint into the sacrum, ensuring optimal immediate stability. Animal studies have confirmed that the microporous structure of 3D-printed materials promotes trabecular bone ingrowth, enhancing long-term stability and preventing prosthesis loosening (48). In the present case, this technology translated into tangible clinical benefits. Preoperative 3D imaging allowed for the precise design and fabrication of the osteotomy guide and prosthesis, thereby enabling accurate implantation and significantly reducing surgical time compared to traditional screw-rod systems. The 3D-printed prosthesis achieved excellent host-implant match and restored symmetrical hip biomechanics, as confirmed by immediate postoperative imaging, which demonstrated symmetrical alignment of the prosthetic and native hip joint rotation centers. Furthermore, the prosthetic screws were securely fixed to the sacrum through the sacroiliac joint without intrusion into the spinal canal, consistent with the preoperative design. The patient achieved ambulation within two weeks postoperatively. At the 12-month follow-up, the patient exhibited satisfactory hip function with no evidence of mechanical failure, local recurrence, or distant metastasis. This outcome aligns with a growing body of evidence, including a retrospective analysis of 35 cases, demonstrating that 3D-printed endoprostheses offer a reliable solution for managing complex pelvic defects, yielding predictable functional outcomes with manageable complications (49). Despite these advantages, the widespread adoption of 3D-printed prostheses can be limited by factors such as cost, the time required for design and manufacturing, and the need for specialized surgical expertise (50). However, in complex cases such as this, the benefits of personalized reconstruction often outweigh these constraints.

In conclusion, this case demonstrates the feasibility and synergistic potential of a novel trimodal strategy—neoadjuvant chemotherapy with anlotinib, DEB-TACE, and 3D-printed reconstruction.

—for managing primary pelvic PUS. To our knowledge, this represents one of the few reported instances demonstrating the successful application of this specific triple-modality combination for pelvic PUS, highlighting its synergistic value in achieving both oncological control and functional restoration in a highly challenging anatomical context. However, the merits of this approach must be balanced against its significant limitations, most notably the substantial toxicity profile observed in our patient. The severe hematological adverse events and hemorrhagic cystitis underscore the aggressive nature of this regimen and the imperative for meticulous patient selection, proactive supportive care, and potential dose optimization in future applications. Furthermore, the reliance on advanced technologies like DEB-TACE and 3D-printing may present challenges regarding cost, technical expertise, and accessibility, potentially limiting its generalizability. Despite these challenges, the synergistic efficacy observed here provides a compelling rationale for further investigation. Future efforts should be directed towards validating this protocol in larger, prospective cohorts to better define its safety and efficacy. Research should also focus on optimizing the sequencing and timing of each therapeutic modality, and exploring strategies to mitigate adverse effects without compromising oncological outcomes. Ultimately, the evolution of such multidisciplinary strategies holds the promise of improving the prognosis and quality of life for patients with these complex and devastating malignancies.

## 4 Conclusions

In summary, this case establishes that an integrated protocol of neoadjuvant chemotherapy with anlotinib, DEB-TACE, and 3D-printed reconstruction represents a feasible and promising strategy for managing complex primary pelvic PUS. This multimodal approach facilitated significant tumor reduction, precise functional restoration, and favorable oncological outcomes. Despite the associated challenges, including treatment-related side effects and the technical demands of 3D-printing, this comprehensive strategy offers a viable therapeutic alternative for similarly complex scenarios and justifies further prospective investigation and clinical validation.

## Data Availability

The original contributions presented in the study are included in the article/**Supplementary Material**. Further inquiries can be directed to the corresponding author.
