# Supplementary figures and images for: Neoadjuvant chemotherapy, DEB-TACE, and 3D-printed prosthesis for primary pelvic pleomorphic undifferentiated sarcoma: a case report
Source: Front Oncol. 2025 Dec 9;15:1641058. doi: 10.3389/fonc.2025.1641058 (PMC12722842; doi:10.3389/fonc.2025.1641058)

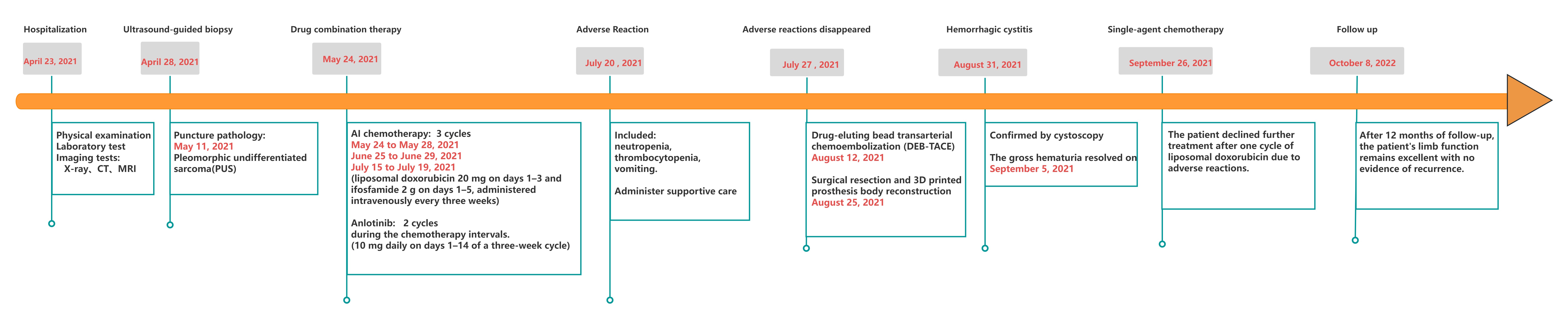

Supplement: Supplementary file 1 [file Image1.jpeg]

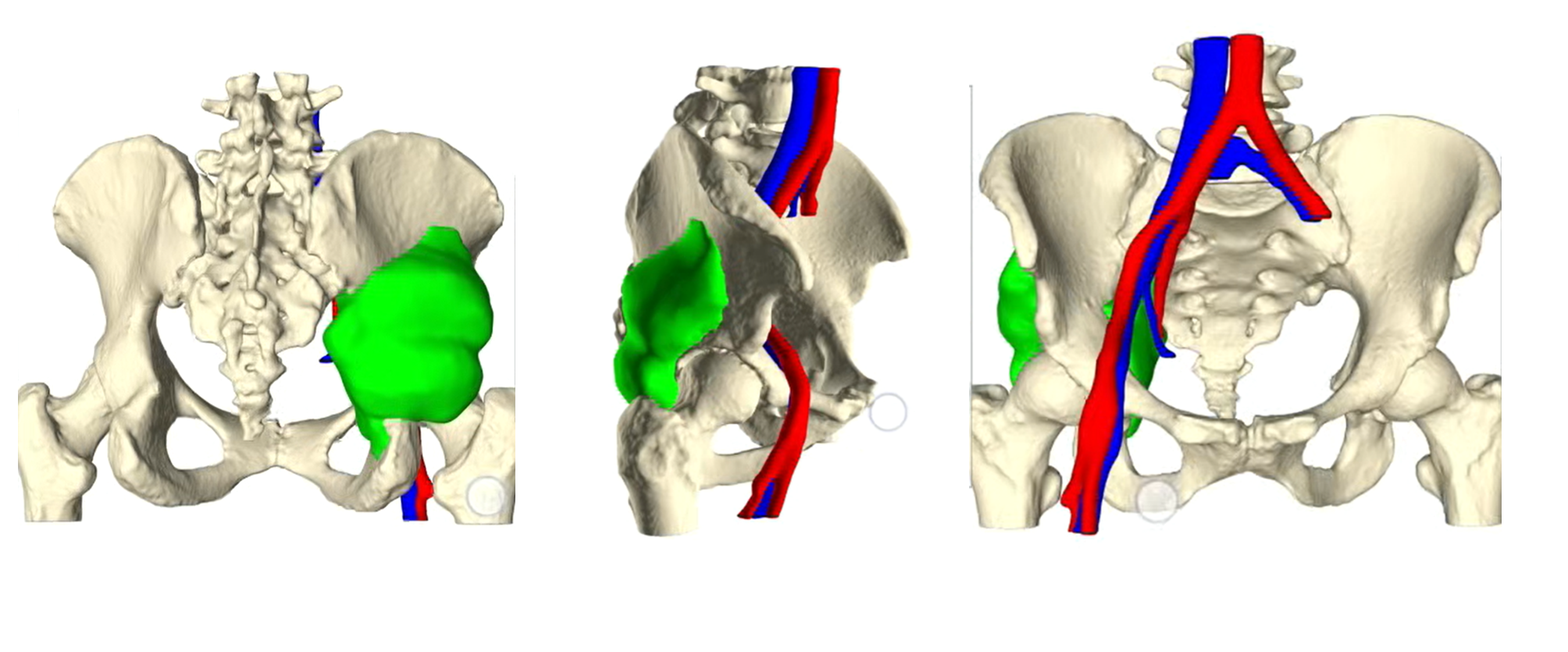

Supplement: Supplementary file 2 [file Image2.tif]
