# Supplementary material for: Neoadjuvant chemotherapy, DEB-TACE, and 3D-printed prosthesis for primary pelvic pleomorphic undifferentiated sarcoma: a case report
Source: Front Oncol. 2025 Dec 9;15:1641058. doi: 10.3389/fonc.2025.1641058 (PMC12722842; doi:10.3389/fonc.2025.1641058)
Supplement: Supplementary file 5 [file Table1.docx]

**Supplementary Figure 1**. Preoperative Radiological Findings

A-C: X-ray and CT before operation：showed a lytic lesion in the right iliac bone and hip joint bone with cortical destruction, surrounding soft tissue mass. D-F: MRI before operation: compared with muscle tissue, showed isointense on T1WI and slightly hyperintense on T2WI, with scattered nodular hyperintensity. The adjacent gluteus and iliopsoas muscle were affected.

**Supplementary Figure 2**. Histopathological Examination of the Tumor

A: Under the microscope, spindle-shaped tumor cells are densely arranged, varying in size, lacking differentiation, and exhibiting significant atypia. The nuclei are large and deeply stained, with prominent nucleoli. Numerous pathological mitotic figures were observed, and the chromatin showed uneven thickness. Additionally, fibroblast cells, histiocyte-like cells, varying numbers of multinucleated giant cells, chronic inflammatory cells, and foam cells were present, consistent with undifferentiated sarcoma. (HE ×200) B: The nuclei of tumor cells vary in size and exhibit irregular shapes. Some of the nuclei display signs of apoptosis, such as chromatin condensation or nuclear fragmentation. The cytoplasm shows vacuolization or eosinophilic change, conform to changes after chemotherapy. (HE×200)

**Supplementary Figure 3.**Post-chemotherapy Imaging and Surgical Intervention

A-F: X-ray, CT and MRI after chemotherapy: showed a significant reduction in tumor volume compared to preoperative images.G: The vascular supply to the tumor was revealed by DEB-TACE. H: X-ray after operation: the fixation of the 3D-printed prosthesis is satisfactory, showing no signs of loosening.

**Supplementary Figure 4.**Twelve-Month Follow-up Imaging

A-F: Follow-up X-ray, CT, and MRI scans after 12 months: no signs of recurrence or metastasis were observed.

**Supplementary Figure 5.**Flowchart.

This figure depicts the relevant workflow and critical timeline of the case.
